# Update on muscle imaging in myositis

**DOI:** 10.1097/BOR.0000000000000975

**Published:** 2023-09-05

**Authors:** Ai Lyn Tan, Andrea Di Matteo, Richard J. Wakefield, John Biglands

**Affiliations:** a Leeds Institute of Rheumatic and Musculoskeletal Medicine, University of Leeds; b NIHR Leeds Biomedical Research Centre, Chapel Allerton Hospital; c Department of Medical Physics & Engineering, Leeds Teaching Hospitals NHS Trust, Leeds, UK

**Keywords:** imaging, MRI, muscle, myositis, ultrasound

## Abstract

**Purpose of review:**

Imaging techniques such as MRI, ultrasound and PET/computed tomography (CT) have roles in the detection, diagnosis and management of myositis or idiopathic inflammatory myopathy (IIM). Imaging research has also provided valuable knowledge in the understanding of the pathology of IIM. This review explores the latest advancements of these imaging modalities in IIM.

**Recent findings:**

Recent advancements in imaging of IIM have seen a shift away from manual and qualitative analysis of the images. Quantitative MRI provides more objective, and potentially more sensitive characterization of fat infiltration and inflammation in muscles. In addition to B-mode ultrasound changes, shearwave elastography offers a new dimension to investigating IIM. PET/CT has the added advantage of including IIM-associated findings such as malignancies.

**Summary:**

It is evident that MRI, ultrasound and PET/CT have important roles in myositis. Continued technological advancement and a quest for more sophisticated applications help drive innovation; this has especially been so of machine learning/deep learning using artificial intelligence and the developing promise of texture analysis.

## INTRODUCTION

Myositis, or idiopathic inflammatory myopathy (IIM), is a heterogenous group of diseases, which involves inflammation of skeletal muscles; these include dermatomyositis, polymyositis and inclusion body myositis (IBM). Basing a diagnosis on clinical history, physical examination and blood tests alone can be insufficient, and therefore other information is often required. Muscle biopsy is invasive and may miss sites of abnormalities. Electromyography (EMG) can be uncomfortable, and the findings may be nonspecific. Imaging techniques therefore offer an alternative means of evaluating muscles, thereby potentially avoiding some of these adverse effects.

In recent years, there has been an increased interest in muscle imaging, driven by a need for early diagnosis and treatment in order to avoid disabling and life-threatening sequelae and the development of new therapeutics. This review explores the latest advances in imaging including quantitative MRI techniques, ultrasonography and PET/computed tomography (PET/CT), which are the three most commonly used imaging tools in the diagnosis and management of IIM. 

**Box 1:**
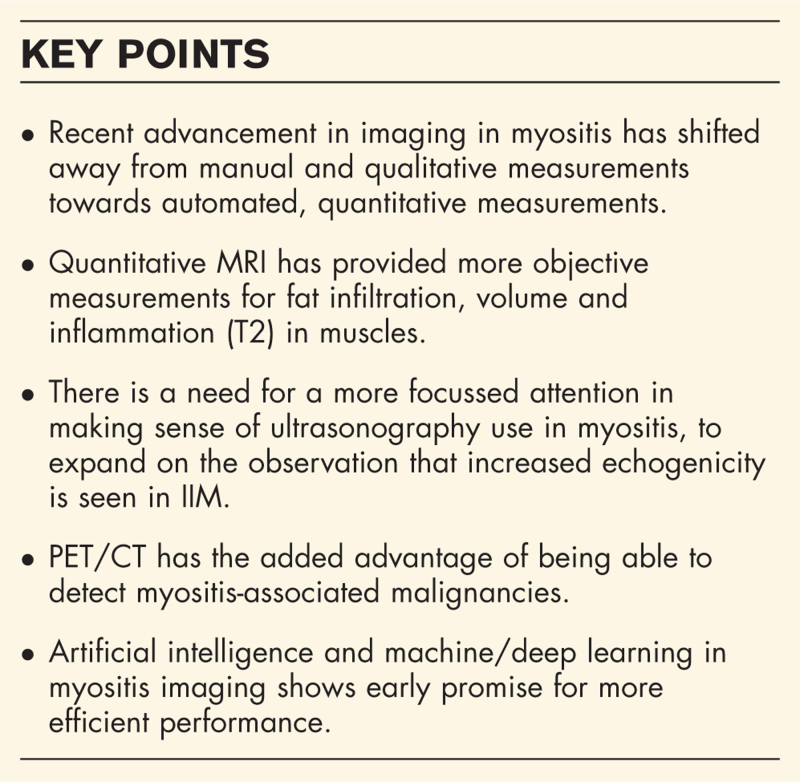
no caption available

## IMAGING IN MYOSITIS

### MRI

MRI is traditionally considered the reference radiological modality for IIM. However, it is limited by its cost, long imaging times and relative lack of availability.

The MRI protocol for IIM typically includes T1 and T2-weighted spin-echo images to visualise fatty replacement, oedema and inflammatory changes, respectively [1]. There are a range of semi-quantitative systems for scoring muscle atrophy, fatty replacement and muscle oedema from MRI, but there is no standardized, validated scoring system for IIM [2]. Existing scoring systems have shown good reproducibility in practice [2]; however, they are subjective, require a skilled observer and depend on the relative differences between healthy and diseased muscles within the same image [3^▪^,^4^]. In comparison, a wide range of quantitative measurements are sensitive to muscle changes in IIM [3^▪^,^5^,^6^] and can detect changes that visual observers miss in isolated studies [3^▪^,^4^].

#### Muscle volume

MRI is the gold-standard technique for measuring muscle volume [7]. Muscle volume is related to muscle function [8,9] and joint torque [10]. Changes in muscle volume can be due to normal physiological causes, for example hypertrophy after training or atrophy due to ageing [11,12] or disease [13,14]. Muscle volume is a difficult measurement to standardize, because so many factors affect it, including exercise, age, sex, height and many more [15]. However, muscle volume has been able to detect differences between IIM and healthy controls [3^▪^], as well as changes due to treatment [16].

Historically, due to the long analysis times, cross-sectional area measurement of muscle on a single slice are often used instead of a 3D muscle volume. This compromise introduces the further potential confounding factor of slice position error. However, a range of automated solutions to the problem of segmenting muscle from MRI have been reported [17–19]. These algorithms significantly reduce the analysis time and are likely to make muscle volume a more readily available tool in the future.

#### Fat fraction

**FIGURE 1:**
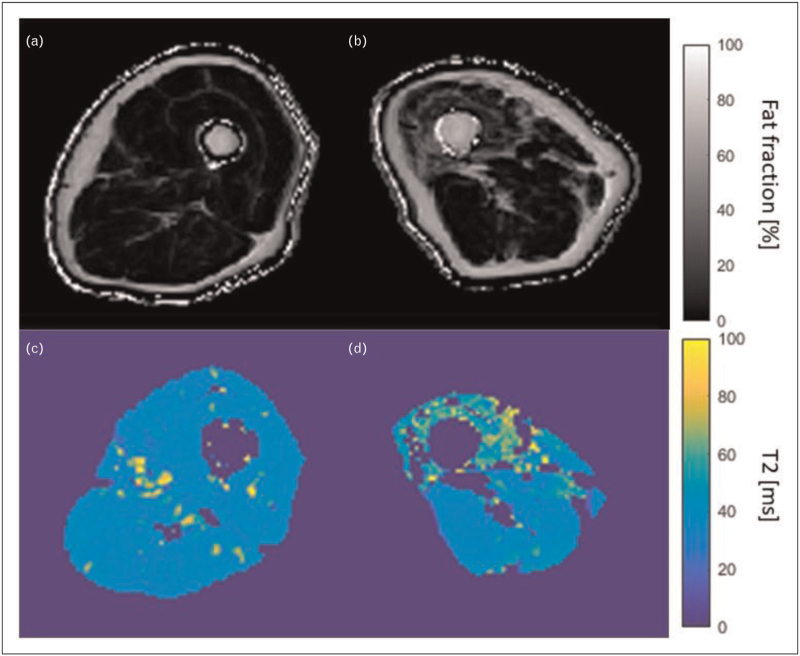
Example of MRI fat fraction (a and b) and T2 maps (c and d) of the thigh muscles in a myositis patient (b and d) as compared to those in a healthy volunteer of similar age and the same sex (a and c). Raised fat content and increased T2 in the quadriceps are indicative of myositis in the patient images.

MRI measurements of intramuscular fat fraction are often used as biomarkers of disease progression and are commonly used as outcome measures in clinical studies [20]. Fat fraction measurements are made using the Dixon techniques, which exploit the fact that fat and water precess at different frequencies in a magnetic field. They use images acquired at carefully chosen echo times, to separate the signals of fat and water so that the fat fraction can be measured. They have been successfully used to quantify fatty infiltration in myositis and to distinguish different levels of fat between muscle groups in individuals [3^▪^,^6^] (Fig. 1). However, IIM is a heterogeneous disease and some patients do not exhibit fatty infiltration. Therefore, although fat fraction is a meaningful tool in the understanding of the disease, it is not specific enough to be used as a stand-alone diagnostic tool.

#### T2 measurements

T2, or transverse relaxation time, is the time constant that determines the rate of transverse signal decay in MRI. An increased T2 can be interpreted as increased fluid content due to oedema or inflammation. T2 values have been shown to be higher in IIM than in healthy muscles [3^▪^,^21^,^22^] (Fig. 1). As a result, T2 measurements show potential as a diagnostic tool for IIM [4,23,24], with some evidence that they can detect abnormalities that semi-quantitative assessments miss [3^▪^] (Table 1).

**Table 1:** Recommendations for the different imaging modality in the management of myositis

	Diagnosis	Monitoring	Intervention	Other
MRI	Ability to identify active muscle inflammation. There is some evidence that quantitative MRI tools may be able to detect disease that radiologists miss.	Useful in monitoring disease both visually and in terms of muscle volume, fat fraction and T2, in distinguishing active muscle inflammation, from mild, low and no inflammation.	Most common imaging modality for identification and selection of regions for muscle biopsy.	The objectivity of quantitative MRI makes it useful for research, with the potential to detect subtle differences in muscle.
Ultrasound	Ability to detect abnormal muscles likely due to IIM, but role is currently unclear.	Changes in muscle echogenicity, intra-muscular power Doppler and muscle stiffness might be useful to assess treatment response (under investigations).	To guide a biopsy needle into muscle often previously highlighted as abnormal by other imaging techniques such as MRI.	Most repeatable imaging tool due to relatively low costs and absence of radiations.
PET-CT	Ability to identify ‘active’ muscle inflammation and, therefore, to distinguish patients with IIM from controls. Sensitivity and specificity values are based on the SUV cut-offs used in the different studies.	Potential ability to distinguish very ‘active’ muscle inflammation, from mild, low and no inflammation, with implications on disease monitoring including response to treatment.	Whole body technique which help identify most ‘active’ regions for muscle biopsy.	Potential ability to screen for cancers, which are relatively common in this population. Good accuracy in the detection of interstitial lung disease.

However, T2 measurements are also subject to a range of potential errors that have been addressed with different acquisitions and analysis strategies. Arguably, the most important of these is the influence of fat on T2 measurements, which can give the impression of heightened water T2 [25]. Fat suppression techniques such as SPAIR supress the fat signal but do not remove it entirely. A number of methods have been proposed to address the issue, but there is no standard solution to the problem [5,26–28]. Therefore, comparisons between T2 measurements made with different imaging systems, sequences or analysis methods should be undertaken with caution.

In summary, although visual assessment of MRI in IIM remains the imaging of choice in clinical practice, quantitative measurements show promise in the potential role in facilitating earlier diagnosis and better capability in monitoring disease progression in IIM, but there is scope for further fine-tuning of the methodologies.

### Ultrasonography

It is only relatively recently that ultrasonography has begun to be considered as a viable tool for investigating patients with IIM, with most previous attention centred around muscle injuries often in the context of sports medicine [29] and neuromuscular disorders [30].

Ultrasonography has a number of advantages when compared to CT and MRI, such as wider availability, the avoidance of radiation (vs. CT) and strong electromagnetic forces (vs. MRI), greater patient acceptability and the allowance of a dynamic assessment. It is, however, not without its own limitations such as restrictions of access to the ultrasonography beam for deeper muscles, and lack of standardization of ultrasonography muscle assessment.

#### Ultrasound appearance of normal muscle

**FIGURE 2:**
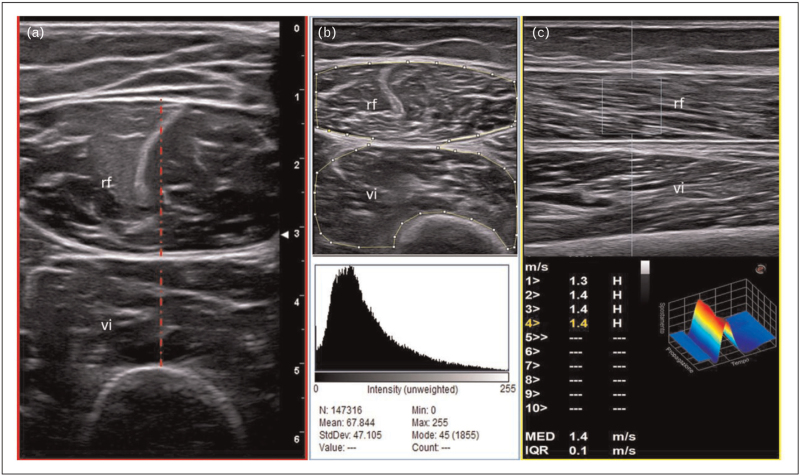
‘Multimodal’ assessment of quadriceps muscle mass (a), muscle quality (b, muscle echogenicity assessment using ImageJ analysis) and muscle stiffness (c, point shear wave elastography) in two healthy individuals. In figure a, the typical ‘starry night’ appearance of a normal muscle (28–year-old man) can be observed. A transverse approach is preferred for the measurement of muscle thickening (red dashed lines) of the rectus femoris (rf) and vastus intermedius (vi) muscles. Figure b shows a moderate increase in muscle echogenicity in a 55-year-old woman. Muscle echogenicity can be measured using a dedicated image analysis program, which measures the gray scale intensity in a region of interest (ROI) utilizing histogram function (i.e. ImageJ). In the same person, muscle stiffness of the rectus femoris is measured using point shear wave elastography (longitudinal approach) and it is expressed by m/s.

Muscle is evaluated using both gray scale (B mode) and Doppler modalities [31]. Supplementary techniques such as elastography have also recently begun to be explored. Broadly, gray scale provides a measure of tissue structure, whilst Doppler evaluates vascularity within it. In contrast, elastography measures the stiffness of tissue (Fig. 2).

Using gray scale, normal muscle appears generally hypoechoic or anechoic relative to surrounding subcutaneous tissue. In longitudinal plane, hyperechoic bands can be seen within the muscle representing the perimysium or aponeurosis. In transverse plane, these bands may give a more ‘dotted’ appearance of the muscle (‘starry night’ appearance). Ultrasonography muscle appearances may differ with respect to the depth of the muscle and type of muscle relating to differences in fibre orientation and size of fibre [32].

The presence of Doppler highlights the position and magnitude of flow within the vessels. It is normal to find blood flow in muscle and fascia using standard ultrasonography equipment.

#### Describing and quantifying muscle disease

**FIGURE 3:**
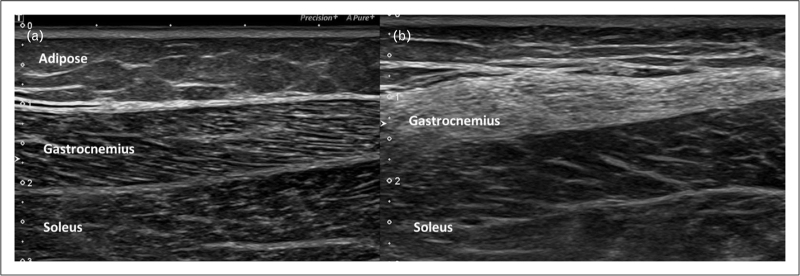
Gray scale longitudinal image through the calf showing. (a) Normal lateral gastrocnemius muscle and soleus muscles. (b) Abnormally hyperechoic gastrocnemius muscle of a 51-year-old woman with IBM. Images Courtesy of Dr Shereen Paramalingam.

A number of authors have reported increased muscle echogenicity in myositis [33] (Fig. 3); however, there are caveats; for example, in the acute phase, oedema as stated previously may serve to decrease muscle echogenicity to either abnormally hypoechoic or normal levels. This oedema may also be associated with blurring of the muscle architecture and increasing muscle thickness [34]. Reassuringly, although not specifically aimed at myositis, a large multicentre study on patients with different rheumatic diseases demonstrated excellent intra-rater and good inter-rater reliability of scoring echogenicity of muscle based on the reading of on-line images and clips [35]. In a follow up study of seven children over 2 years reported that after the administration of prednisolone, the echogenicity of the muscle increased, presumed due to the loss of oedema [34].

Several attempts have been made to quantify muscle using ultrasonography. This has been on the basis of muscle thickness, cross-sectional area or its qualitative appearance. Heckmatt *et al.*[36], for example, developed a qualitative 4-point scale (1–4) based on increased degrees of muscle echogenicity. The authors noted that changes in muscle may be focal or diffuse. Quantitative ‘computer assisted’ gray scale measurements may be made with software, which evaluates the total hue within a region of interest (ROI). Recently, di Matteo *et al.*[37^▪^] developed a modified version of the Heckmatt scale, measuring grades of echointensity abnormalities based on the extent of muscle involved. However, relatively increased connective tissue due to muscle atrophy can appear to increase the echointensity, so this modified scale will need to be further validated.

An important early study investigating the role of ultrasonography in myositis evaluated a range of biopsy-proven myositis cases as well as a large normal control group [38]. In the patients, ROI and visual measurements and biopsy result correlated in 58 out of 70 (83%) patients. Interestingly, this study suggested a correlation between fatty infiltration and increased echogenicity rather than muscle fibrosis *per se*. However, it also concluded that the more chronic the myositis, the more echogenic the muscle, and that conversely those with more oedematous muscle had a slightly less echogenic appearance. It should be remembered that when this study was undertaken, technology and muscle treatments were less advanced, with steroids being the main treatment.

Muscle thickness appears to increase in acute myositis and diminish over time [39] using a number of different measurement techniques. Some authors have considered fascial thickness [40] and perimyseal septal counts [41] as items to evaluate, but the data are small and definitions uncertain. A few studies have evaluated vascularity of muscle with only one using a clear scoring system. In this study of 37 IIM patients, Doppler appeared to be increased in early cases and then decreased over time, in contrast to the gray scale echogenicity, which increased over time [42].

Little data are available for the longitudinal assessment of disease activity: One study in 11 patients with IIM demonstrated changes in echogenicity measures over a 6-month period [43]. Two reported studies in paediatric patients have highlighted changes in muscle echogenicity, which appear to normalize with treatment, although this may take 6–12 months [34,44]. More recently, Walter *et al.*[45^▪▪^] demonstrated that ultrasonography was able to detect changes in as little as 9 weeks.

New tools hold some promise for improving muscle assessment; however, there remains limited data. Elastography, for example, has demonstrated a reduction in muscle stiffness in some studies [46,47^▪▪^], whilst others have shown a greater stiffness [48]. This may reflect the different methods of measurement, the small numbers of patients being evaluated or a relationship with different treatments. Contrast-enhanced ultrasound has been shown to correlate with MRI oedema in patients with histological defined myositis [49]. However, this will be limited by feasibility in practice.

In summary, although the use of ultrasonography for muscle is longstanding, there remain little data on IIM. To some extent, research has been hampered by the rarity of the diseases. Perhaps because of this, there has been a lack of standardization and a previous lack of therapeutic options. As a result, recent attention from groups such as OMERACT have begun to systematically study the technique, and through standardization, multicentre studies may offer the better prospect of an improved understanding.

### PET/computed tomography

PET combined with CT, ‘hybrid PET/CT’ has a promising role in the diagnosis and management of IIM patients. In IIM patients, PET/CT scan offers the possibility to assess muscle structural changes and inflammatory activity simultaneously, usually using F-18 fluorodeoxyglucose (FDG) as a tracer.

A very recent systematic literature review has described the studies exploring the clinical usefulness of PET/CT in IIM patients [50]. We will focus on the value of PET/CT in the diagnosis and assessment of disease activity.

The first study evaluating PET (without CT) in IIM patients was carried out by Owada *et al.*[51]. In this study, an increased FDG muscle uptake was found in eight out of 24 (33%) patients with ‘active’ myositis (11polymyositis and 13 dermatomyositis) and two out of 69 (3%) controls. Therefore, although the specificity of PET was very high, sensitivity was low and significantly lower than electromyography, MRI and muscle biopsy.

Subsequently, Pipitone *et al.*[52] found a higher FDG muscle intake in IIM patients than in disease controls (median 0.58 vs. 0.30, *P* < 0.001) in another small cohort. Using a muscle/liver standardized uptake values (SUV) ratio of 0.45, the sensitivity/specificity of PET/CT was 75 and 100%, respectively. In this study, no significant correlation between the PET/CT and creatine kinase (CK) levels, muscle strength and MRI was found.

Tanaka *et al.*[53] also showed a higher FDG muscle uptake in IIM patients than in controls (median 1.05 vs. 0.69, *P* < 0.001). When using a SUV cut-off of 0.83, the sensitivity and specificity of PET/CT for IIM were 90 and 100%, respectively. Interestingly, SUV values more than 0.83 were found in three out of eight IIM patients with a normal MRI scan, thus suggesting a higher sensitivity of PET/CT in at least some patients. Unlike the study by Pipitone *et al.* [52], a significant association between PET/CT and reduced patients’ muscle strength and serum CK/aldolase levels was reported. In addition, higher SUV values in proximal muscles correlated with inflammation on histology, thus highlighting the value of PET/CT as a guide for the selection of the regions for muscle biopsy in IIM patients.

Similar positive results were obtained in two retrospective studies, in which PET/CT was able to discriminate between IIM patients and controls [54,55].

Recently, Matuszak *et al.*[56] proposed PET-CT as an instrument that could be used for monitoring disease activity in IIM patients. Using the Myositis Intention to Treat Activity Index (which scores ‘disease activity’ from active to inactive in a muscle domain), the authors identified a SUV threshold of 0.66, which was able to differentiate high muscle disease activity from low or no muscle with a high sensitivity and specificity.

The main application for PET/CT in clinical practice is to investigate the presence of a malignancy. The strong association between IIM and cancers is well known [57]. Given the increased cancers rate in IIM patients, early screening and surveillance for malignancy is recommended in IIM patients.

International guidelines on cancer screening in patients with IIM are lacking [58]. Furthermore, whether PET/CT offers any additional value compared with a routine battery of screening tests for neoplasm in IIM patients remains undefined. The studies that have explored this aspect showed a sensitivity and specificity of PET/CT of 66.7–94 and 80–97.8%, respectively [59–62].

In addition, PET-CT has been shown to be accurate for the assessment of interstitial lung disease (a common manifestation in patients with IIM), with a comparable sensitivity (93–100%) to high-resolution CT, which is regarded as the gold standard for the assessment of this condition [63–65].

In summary, PET/CT showed a very high specificity and good sensitivity for the diagnosis and assessment of disease activity in IIM patients. PET/CT can detect cancer (which is relatively common in this population) and is able to assess ‘extra-muscular’ targets of IIM, such as the lung. PET/CT is a whole-body technique, which could help guiding the selection of the regions for muscle biopsy.

Main limitations are patients’ radiation exposure, costs and availability. Further research comparing the accuracy of PET/CT, MRI and ultrasonography for the diagnosis and follow-up (i.e. responsiveness to treatment) of IIM patients are needed to understand the ‘true’ clinical usefulness of PET/CT in the assessment of these patients [66^▪^].

## FUTURE DIRECTIONS

Although MRI remains the main imaging tool in the clinical management of IIM, there is potential for quantitative MRI to be a more sensitive and accurate tool in detecting subtle myositic changes. The use of imaging in disease management lends itself well to being enhanced by artificial intelligence (AI). AI offers the potential to make imaging more streamlined and lean from the perspective of productivity, time and resource, as well as being more reliable in detecting abnormalities. Machine learning has been shown to automate processes such as measuring body composition, which includes muscle and fat mass [67]. Deep learning algorithms have been applied to muscle segmentation (to assess muscle volume and localize muscles for quantitative analysis), and have been shown to be more accurate than manual segmentation in preparation for surgery [68]. More importantly, deep learning can be trained to differentiate between myopathies, and is thereby potentially useful in the diagnosis of IIM [69^▪^].

As we progress in our learning of the potential of AI in enhancing the use of imaging in detecting abnormalities, our research methods become more sophisticated. For example, a fully automated deep learning algorithm for diagnosing myositis using ultrasonography has been shown to perform better and more accurately than semi-automated machine learning techniques that require manual delineation of muscle and fat boundaries [70,71].

As alluded to earlier, increased ultrasound echogenicity has been observed in muscles involved in myositis. Texture analysis based on pixel-based echogenicity of an ultrasound image is a technique that attempts to differentiate the various myopathies using mathematical analysis of muscular microstructure not visible to the human eye. The technique has been shown to be able to classify different myopathies [72]. Unsurprisingly, other imaging modalities have used the application of texture analysis, such as MRI which has been used to predict specific groups of IIM [73].

This could be an important direction on the research agenda, because if this proves to be feasible, reliable and repeatable, it may mean that patients with IIM can avoid unnecessary and invasive muscle biopsies for diagnosis and management of their conditions.

## CONCLUSION

Muscle imaging in IIM has stretched the potential of various imaging modalities such as MRI, ultrasonography and PET/CT in detecting and diagnosing myositis, and has become an important part of the management of IIM. Yet, the recent research in this area suggests that there is still untapped exploitation of the various imaging techniques, coupled with AI, which could continue to revolutionize the use of imaging in myositis.

## Acknowledgements

*None.*

### Financial support and sponsorship

*None.*

### Conflicts of interest

*There are no conflicts of interest.*

## REFERENCES AND RECOMMENDED READING

Papers of particular interest, published within the annual period of review, have been highlighted as:

▪ of special interest
▪▪ of outstanding interest

## References

[1] Albayda J Demonceau G Carlier PG. Muscle imaging in myositis: MRI, US, and PET. Best Pract Res Clin Rheumatol 2022; 36:101765. 35760742 10.1016/j.berh.2022.101765

[2] Barsotti S Zampa V Talarico R. Thigh magnetic resonance imaging for the evaluation of disease activity in patients with idiopathic inflammatory myopathies followed in a single center. Muscle Nerve 2016; 54:666–672. 27279002 10.1002/mus.25099

[3▪] Farrow M Biglands JD Grainger AJ. Quantitative MRI in myositis patients: comparison with healthy volunteers and radiological visual assessment. Clin Radiol 2021; 76:81.e1–81.e10. 10.1016/j.crad.2020.08.022 32958223

[4] Carlier PG Azzabou N de Sousa PL. P.14.4 Diagnostic role of quantitative NMR imaging exemplified by 3 cases of juvenile dermatomyositis. Neuromuscular Disord 2013; 23:814.

[5] Yao L Yip AL Shrader JA. Magnetic resonance measurement of muscle T2, fat-corrected T2 and fat fraction in the assessment of idiopathic inflammatory myopathies. Rheumatology (Oxford) 2016; 55:441–449. 26412808 10.1093/rheumatology/kev344 PMC4757924

[6] Sigmund EE Baete SH Luo T. MRI assessment of the thigh musculature in dermatomyositis and healthy subjects using diffusion tensor imaging, intravoxel incoherent motion and dynamic DTI. Eur Radiol 2018; 28:5304–5315. 29869178 10.1007/s00330-018-5458-3 PMC11980643

[7] Pons C Borotikar B Garetier M. Quantifying skeletal muscle volume and shape in humans using MRI: a systematic review of validity and reliability. PLoS One 2018; 13:e0207847. 30496308 10.1371/journal.pone.0207847 PMC6264864

[8] Trappe SW Trappe TA Lee GA Costill DL. Calf muscle strength in humans. Int J Sports Med 2001; 22:186–191. 11354521 10.1055/s-2001-16385

[9] Lieber RL Fridén J. Functional and clinical significance of skeletal muscle architecture. Muscle Nerve 2000; 23:1647–1666. 11054744 10.1002/1097-4598(200011)23:11<1647::aid-mus1>3.0.co;2-m

[10] Fukunaga T Miyatani M Tachi M. Muscle volume is a major determinant of joint torque in humans. Acta Physiol Scand 2001; 172:249–255. 11531646 10.1046/j.1365-201x.2001.00867.x

[11] Farrow M Biglands J Tanner SF. The effect of ageing on skeletal muscle as assessed by quantitative MR imaging: an association with frailty and muscle strength. Aging Clin Exp Res 2021; 33:291–301. 32198628 10.1007/s40520-020-01530-2 PMC7914187

[12] Narici MV Maganaris CN Reeves ND Capodaglio P. Effect of aging on human muscle architecture. J Appl Physiol 2003; 95:2229–2234. 12844499 10.1152/japplphysiol.00433.2003

[13] Marcon M Ciritsis B Laux C. Cross-sectional area measurements versus volumetric assessment of the quadriceps femoris muscle in patients with anterior cruciate ligament reconstructions. Eur Radiol 2015; 25:290–298. 25358592 10.1007/s00330-014-3424-2

[14] Pons C Sheehan FT Im HS. Shoulder muscle atrophy and its relation to strength loss in obstetrical brachial plexus palsy. Clin Biomech (Bristol, Avon) 2017; 48:80–87. 28783492 10.1016/j.clinbiomech.2017.07.010 PMC5628613

[15] Hogrel JY Barnouin Y Azzabou N. NMR imaging estimates of muscle volume and intramuscular fat infiltration in the thigh: variations with muscle, gender, and age. Age (Dordr) 2015; 37:9798. 26040416 10.1007/s11357-015-9798-5 PMC4456487

[16] Amato AA Sivakumar K Goyal N. Treatment of sporadic inclusion body myositis with bimagrumab. Neurology 2014; 83:2239–2246. 25381300 10.1212/WNL.0000000000001070 PMC4277670

[17] Friedberger A Figueiredo C Bäuerle T. A new method for quantitative assessment of hand muscle volume and fat in magnetic resonance images. BMC Rheumatol 2020; 4:72. 33349274 10.1186/s41927-020-00170-3 PMC7754591

[18] Karlsson A Rosander J Romu T. Automatic and quantitative assessment of regional muscle volume by multiatlas segmentation using whole-body water-fat MRI. J Magn Reson Imaging 2015; 41:1558–1569. 25111561 10.1002/jmri.24726

[19] Middleton MS Haufe W Hooker J. Quantifying abdominal adipose tissue and thigh muscle volume and hepatic proton density fat fraction: repeatability and accuracy of an MR imaging-based, semiautomated analysis method. Radiology 2017; 283:438–449. 28278002 10.1148/radiol.2017160606 PMC5410959

[20] Carlier PG Marty B Scheidegger O. Skeletal muscle quantitative nuclear magnetic resonance imaging and spectroscopy as an outcome measure for clinical trials. J Neuromuscul Dis 2016; 3:1–28. 27854210 10.3233/JND-160145 PMC5271435

[21] Maillard SM Jones R Owens C. Quantitative assessment of MRI T2 relaxation time of thigh muscles in juvenile dermatomyositis. Rheumatology (Oxford) 2004; 43:603–608. 14983103 10.1093/rheumatology/keh130

[22] Ran J Ji S Morelli JN. T2 mapping in dermatomyositis/polymyositis and correlation with clinical parameters. Clin Radiol 2018; 73:1057.e13–1057.e18. 10.1016/j.crad.2018.07.106 30172348

[23] Ran J Ji S Morelli JN. The diagnostic value of T(2) maps and rs-EPI DWI in dermatomyositis. Br J Radiol 1094; 92:20180715. 10.1259/bjr.20180715 PMC6404843 30383453

[24] Huber AT Bravetti M Lamy J. Noninvasive differentiation of idiopathic inflammatory myopathy with cardiac involvement from acute viral myocarditis using cardiovascular magnetic resonance imaging T1 and T2 mapping. J Cardiovasc Magn Reson 2018; 20:11. 29429407 10.1186/s12968-018-0430-6 PMC5808400

[25] Carlier PG. Global T2 versus water T2 in NMR imaging of fatty infiltrated muscles: different methodology, different information and different implications. Neuromuscul Disord 2014; 24:390–392. 24656605 10.1016/j.nmd.2014.02.009

[26] Marty B Baudin PY Reyngoudt H. Simultaneous muscle water T2 and fat fraction mapping using transverse relaxometry with stimulated echo compensation. NMR Biomed 2016; 29:431–443. 26814454 10.1002/nbm.3459

[27] Ababneh Z Beloeil H Berde CB. Biexponential parameterization of diffusion and T2 relaxation decay curves in a rat muscle edema model: decay curve components and water compartments. Magn Reson Med 2005; 54:524–531. 16086363 10.1002/mrm.20610

[28] Azzabou N Loureiro de Sousa P Caldas E Carlier PG. Validation of a generic approach to muscle water T2 determination at 3T in fat-infiltrated skeletal muscle. J Magn Reson Imaging 2015; 41:645–653. 24590466 10.1002/jmri.24613

[29] Paoletta M Moretti A Liguori S. Ultrasound imaging in sport-related muscle injuries: pitfalls and opportunities. Medicina 2021; 57:1040. 34684077 10.3390/medicina57101040 PMC8540210

[30] Heckmatt JZ Dubowitz V Leeman S. Detection of pathological change in dystrophic muscle with B-scan ultrasound imaging. Lancet 1980; 1:1389–1390. 6104175 10.1016/s0140-6736(80)92656-2

[31] Pillen S Boon A Van Alfen N. Muscle ultrasound. Handb Clin Neurol 2016; 136:843–853. 27430445 10.1016/B978-0-444-53486-6.00042-9

[32] Van Holsbeeck M Introcaso J. Van Holsbeeck M Introcaso J. Sonography of muscle. Musculoskeletal ultrasound 2nd ed. St Louis, MO: Mosby; 2001. 23–75.

[33] Paramalingam S Morgan K Becce F. Conventional ultrasound and elastography as imaging outcome tools in autoimmune myositis: a systematic review by the OMERACT ultrasound group. Semin Arthritis Rheum 2021; 51:661–676. 33386164 10.1016/j.semarthrit.2020.11.001

[34] Habers GE Van Brussel M Bhansing KJ. Quantitative muscle ultrasonography in the follow-up of juvenile dermatomyositis. Muscle Nerve 2015; 52:540–546. 25557638 10.1002/mus.24564

[35] Di Matteo A Moscioni E Lommano MG. Reliability assessment of ultrasound muscle echogenicity in patients with rheumatic diseases: results of a multicenter international web-based study. Front Med (Lausanne) 2022; 9:1090468. 36733934 10.3389/fmed.2022.1090468 PMC9886677

[36] Heckmatt JZ Leeman S Dubowitz V. Ultrasound imaging in the diagnosis of muscle disease. J Pediatr 1982; 101:656–660. 7131136 10.1016/s0022-3476(82)80286-2

[37▪] Di Matteo A Smerilli G Cipolletta E. Muscle involvement in systemic lupus erythematosus: multimodal ultrasound assessment and relationship with physical performance. Rheumatology (Oxford) 2022; 61:4775–4785. 35333315 10.1093/rheumatology/keac196

[38] Reimers CD Fleckenstein JL Witt TN. Muscular ultrasound in idiopathic inflammatory myopathies of adults. J Neurol Sci 1993; 116:82–92. 8509807 10.1016/0022-510x(93)90093-e

[39] Kuo GP Carrino JA. Skeletal muscle imaging and inflammatory myopathies. Curr Opin Rheumatol 2007; 19:530–535. 17917531 10.1097/BOR.0b013e3282efdc66

[40] Bhansing KJ Van Rosmalen MH Van Engelen BG. Increased fascial thickness of the deltoid muscle in dermatomyositis and polymyositis: an ultrasound study. Muscle Nerve 2015; 52:534–539. 25655014 10.1002/mus.24595

[41] Leeuwenberg K Albayda J. Muscle ultrasound in inflammatory myopathies: a critical review. J Rheum Dis Treat 2019; 5:

[42] Meng C Adler R Peterson M Kagen L. Combined use of power Doppler and gray-scale sonography: a new technique for the assessment of inflammatory myopathy. J Rheumatol 2001; 28:1271–1282. 11409119

[43] Mittal GA Wadhwani R Shroff M. Ultrasonography in the diagnosis and follow-up of idiopathic inflammatory myopathies--a preliminary study. J Assoc Physicians India 2003; 51:252–256. 12839345

[44] Bhansing KJ Hoppenreijs EP Janssen AJ. Quantitative muscle ultrasound: a potential tool for assessment of disease activity in juvenile dermatomyositis. Scand J Rheumatol 2014; 43:339–341. 24720507 10.3109/03009742.2013.879674

[45▪▪] Walter AW Lim J Raaphorst J. Ultrasound and MR muscle imaging in new onset idiopathic inflammatory myopathies at diagnosis and after treatment: a comparative pilot study. Rheumatology (Oxford) 2022; 62:300–309. 35536176 10.1093/rheumatology/keac263 PMC9788821

[46] Alfuraih AM O’Connor P Tan AL. Muscle shear wave elastography in idiopathic inflammatory myopathies: a case-control study with MRI correlation. Skeletal Radiol 2019; 48:1209–1219. 30810778 10.1007/s00256-019-03175-3 PMC6584706

[47▪▪] Paramalingam S Needham M Raymond W. Muscle shear wave elastography, conventional B mode and power doppler ultrasonography in healthy adults and patients with autoimmune inflammatory myopathies: a pilot cross-sectional study. BMC Musculoskelet Disord 2021; 22:537. 34118902 10.1186/s12891-021-04424-0 PMC8199828

[48] Kolb M Ekert K Schneider L. The utility of shear-wave elastography in the evaluation of myositis. Ultrasound Med Biol 2021; 47:2176–2185. 34030894 10.1016/j.ultrasmedbio.2021.04.010

[49] Weber MA Jappe U Essig M. Contrast-enhanced ultrasound in dermatomyositis- and polymyositis. J Neurol 2006; 253:1625–1632. 17219033 10.1007/s00415-006-0318-5

[50] Bentick G Fairley J Nadesapillai S. Defining the clinical utility of PET or PET-CT in idiopathic inflammatory myopathies: a systematic literature review. Semin Arthritis Rheum 2022; 57:152107. 36335683 10.1016/j.semarthrit.2022.152107

[51] Owada T Maezawa R Kurasawa K. Detection of inflammatory lesions by f-18 fluorodeoxyglucose positron emission tomography in patients with polymyositis and dermatomyositis. J Rheumatol 2012; 39:1659–1665. 22753657 10.3899/jrheum.111597

[52] Pipitone N Versari A Zuccoli G. 18F-Fluorodeoxyglucose positron emission tomography for the assessment of myositis: a case series. Clin Exp Rheumatol 2012; 30:570–573. 22703951

[53] Tanaka S Ikeda K Uchiyama K. [18F]FDG uptake in proximal muscles assessed by PET/CT reflects both global and local muscular inflammation and provides useful information in the management of patients with polymyositis/dermatomyositis. Rheumatology (Oxford) 2013; 52:1271–1278. 23479721 10.1093/rheumatology/ket112

[54] Tateyama M Fujihara K Misu T. Clinical values of FDG PET in polymyositis and dermatomyositis syndromes: imaging of skeletal muscle inflammation. BMJ Open 2015; 5:e006763. 10.1136/bmjopen-2014-006763 PMC4298089 25582454

[55] Arai-Okuda H Norikane T Yamamoto Y. (18)F-FDG PET/CT in patients with polymyositis/dermatomyositis: correlation with serum muscle enzymes. Eur J Hybrid Imaging 2020; 4:14. 34191182 10.1186/s41824-020-00084-w PMC8218055

[56] Matuszak J Blondet C Hubelé F. Muscle fluorodeoxyglucose uptake assessed by positron emission tomography-computed tomography as a biomarker of inflammatory myopathies disease activity. Rheumatology 2019; 58:1459–1464. 10.1093/rheumatology/kez040 30851092

[57] Opinc AH Makowska JS. Update on malignancy in myositis-well established association with unmet needs. Biomolecules 2022; 12:111. 35053259 10.3390/biom12010111 PMC8773676

[58] Oldroyd AGS Allard AB Callen JP. A systematic review and meta-analysis to inform cancer screening guidelines in idiopathic inflammatory myopathies. Rheumatology (Oxford) 2021; 60:2615–2628. 33599244 10.1093/rheumatology/keab166 PMC8213426

[59] Berner U Menzel C Rinne D. Paraneoplastic syndromes: detection of malignant tumors using [(18)F]FDG-PET. Q J Nucl Med 2003; 47:85–89. 12865868

[60] Trallero-Araguás E Gil-Vila A Martínez-Gómez X. Cancer screening in idiopathic inflammatory myopathies: ten years experience from a single center. Semin Arthritis Rheum 2022; 53:151940. 35051890 10.1016/j.semarthrit.2021.12.008 PMC11678788

[61] Li X Tan H. Value of (18)F-FDG PET/CT in the detection of occult malignancy in patients with dermatomyositis. Heliyon 2020; 6:e03707. 32274435 10.1016/j.heliyon.2020.e03707 PMC7132068

[62] Selva-O’Callaghan A Grau JM Gámez-Cenzano C. Conventional cancer screening versus PET/CT in dermatomyositis/polymyositis. Am J Med 2010; 123:558–562. 20569766 10.1016/j.amjmed.2009.11.012

[63] Hervier B Uzunhan Y. Inflammatory myopathy-related interstitial lung disease: from pathophysiology to treatment. Front Med (Lausanne) 2019; 6:326. 32010700 10.3389/fmed.2019.00326 PMC6978912

[64] Li Y Zhou Y Wang Q. Multiple values of (18)F-FDG PET/CT in idiopathic inflammatory myopathy. Clin Rheumatol 2017; 36:2297–2305. 28831580 10.1007/s10067-017-3794-3

[65] Motegi SI Fujiwara C Sekiguchi A. Clinical value of (18) F-fluorodeoxyglucose positron emission tomography/computed tomography for interstitial lung disease and myositis in patients with dermatomyositis. J Dermatol 2019; 46:213–218. 30614031 10.1111/1346-8138.14758

[66▪] Girija MS Tiwari R Vengalil S. PET-MRI in idiopathic inflammatory myositis: a comparative study of clinical and immunological markers with imaging findings. Neurol Res Pract 2022; 4:49. 36210472 10.1186/s42466-022-00213-9 PMC9549636

[67] Wang B Torriani M. Artificial intelligence in the evaluation of body composition. Semin Musculoskelet Radiol 2020; 24:30–37. 31991450 10.1055/s-0039-3400267

[68] Medina G Buckless CG Thomasson E. Deep learning method for segmentation of rotator cuff muscles on MR images. Skeletal Radiol 2021; 50:683–692. 32939590 10.1007/s00256-020-03599-2

[69▪] Fabry V Mamalet F Laforet A. A deep learning tool without muscle-by-muscle grading to differentiate myositis from facio-scapulo-humeral dystrophy using MRI. Diagn Interv Imaging 2022; 103:353–359. 35292217 10.1016/j.diii.2022.01.012

[70] Burlina P Billings S Joshi N Albayda J. Automated diagnosis of myositis from muscle ultrasound: exploring the use of machine learning and deep learning methods. PLoS One 2017; 12:e0184059. 28854220 10.1371/journal.pone.0184059 PMC5576677

[71] Burlina P Joshi N Billings S. Deep embeddings for novelty detection in myopathy. Comput Biol Med 2019; 105:46–53. 30583249 10.1016/j.compbiomed.2018.12.006

[72] Nodera H Sogawa K Takamatsu N. Texture analysis of sonographic muscle images can distinguish myopathic conditions. J Med Invest 2019; 66:237–247. 31656281 10.2152/jmi.66.237

[73] Nagawa K Suzuki M Yamamoto Y. Texture analysis of muscle MRI: machine learning-based classifications in idiopathic inflammatory myopathies. Sci Rep 2021; 11:9821. 33972636 10.1038/s41598-021-89311-3 PMC8110584

